# Optimization of the fused deposition modeling-based fabrication process for polylactic acid microneedles

**DOI:** 10.1038/s41378-021-00284-9

**Published:** 2021-08-02

**Authors:** Libo Wu, Jongho Park, Yuto Kamaki, Beomjoon Kim

**Affiliations:** Institute of Industrial Science, The University of Tokyo, 4-6-1 Komaba, Meguro-ku, Tokyo, 153-8505 Japan

**Keywords:** Engineering, Materials science, Structural properties

## Abstract

A microneedle (MN) array is a novel biomedical device adopted in medical applications to pierce through the stratum corneum while targeting the viable epidermis and dermis layers of the skin. Owing to their micron-scale dimensions, MNs can minimize stimulations of the sensory nerve fibers in the dermis layer. For medical applications, such as wound healing, biosensing, and drug delivery, the structure of MNs significantly influences their mechanical properties. Among the various microfabrication methods for MNs, fused deposition modeling (FDM), a commercial 3D printing method, shows potential in terms of the biocompatibility of the printed material (polylactic acid (PLA)) and preprogrammable arbitrary shapes. Owing to the current limitations of FDM printer resolution, conventional micron-scale MN structures cannot be fabricated without a post-fabrication process. Hydrolysis in an alkaline solution is a feasible approach for reducing the size of PLA needles printed via FDM. Moreover, weak bonding between PLA layers during additive manufacturing triggers the detachment of PLA needles before etching to the expected sizes. Furthermore, various parameters for the fabrication of PLA MNs with FDM have yet to be sufficiently optimized. In this study, the thermal parameters of the FDM printing process, including the nozzle and printing stage temperatures, were investigated to bolster the interfacial bonding between PLA layers. Reinforced bonding was demonstrated to address the detachment challenges faced by PLA MNs during the chemical etching process. Furthermore, chemical etching parameters, including the etchant concentration, environmental temperature, and stirring speed of the etchant, were studied to determine the optimal etching ratio. To develop a universal methodology for the batch fabrication of biodegradable MNs, this study is expected to optimize the conditions of the FDM-based fabrication process. Additive manufacturing was employed to produce MNs with preprogrammed structures. Inclined MNs were successfully fabricated by FDM printing with chemical etching. This geometrical structure can be adopted to enhance adhesion to the skin layer. Our study provides a useful method for fabricating MN structures for various biomedical applications.

## Introduction

A commercially available microneedle (MN) patch is primarily a cosmetic product that contains micron-sized polymer needles. The concept of MNs was first introduced in 1976 for transdermal drug delivery^1^. Biomedical applications of MNs have been further developed for wound healing and biosensing^2–4^. MNs can bypass the stratum corneum, which is the outermost barrier layer of the skin. Active pharmaceutical ingredients (APIs) are subsequently administered to the viable epidermis and dermis layers of the skin. MN-mediated applications enable delivered APIs to block abnormal cell cycles in the skin, which can treat lesions^5^. Compared with the systemic drug delivery method, drug delivery by MNs targeting affected dermal cells can improve the efficiency of drug administration and eliminate drug side effects^6^. The physical stimulation of epithelial and endothelial cells in wounded areas has been demonstrated to induce the genetic expression of growth factors, which facilitate wound healing^7^.

There has been increasing interest in MN-mediated biosensing. The targets of MN-mediated biosensing are blood or interstitial fluid (ISF). ISF in the epidermis is a source of several biomarkers, such as ions, proteins, metabolites, and glucose. MNs can be used to painlessly collect biomarkers from ISFs in the epidermis. Biosensing platforms that significantly minimize the invasiveness of the detection of several analytes have been considered for various MN-based diagnoses^8^.

Various MN fabrication methods have been researched, such as molding methods and drawing lithography^9,10^. Among them, molding is the most conventionally adopted fabrication method for polymer MNs. However, demolding MNs from negative molds can potentially destroy the structures of MNs. Wearable biomedical applications of MNs have been considered, and the geometrical structures of MNs have been recently investigated for targeted administration on typical skin layers and strong adhesions to the skin^11^. Additive manufacturing (AM) is a construction method for object development using digital three-dimensional (3D) models. AM methods, such as digital light processing (DLP), stereolithography (SLA), and fused deposition modeling (FDM), are novel candidates for the fabrication of polymer MNs. Owing to the bottom–up approach, AM methods can be used to fabricate MNs with predesigned structures, which cannot be realized via the molding method.

**Fig. 1:**
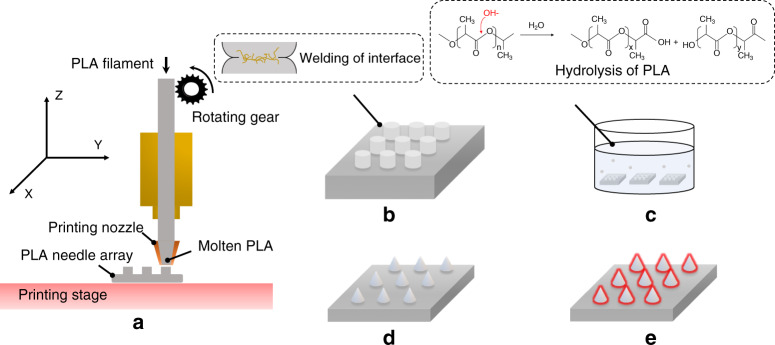
Illustration of PLA MNs fabricated by combining FDM and chemical etching. **a** PLA needle arrays with predesigned structures printed by FDM. **b** PLA needle arrays printed under the optimized condition to form optimal welding at the interfaces of successive layers. **c** PLA needles chemically etched via the hydrolysis of PLA under alkaline conditions. **d** PLA MN array obtained after chemical etching. **e** PLA MNs coated with API

DLP and SLA methodologies have been adopted to fabricate photopolymer MNs with high resolution^12,13^. The potential skin sensitization triggered by the photopolymers used in DLP and SLA is non-negligible. The safety of the photopolymers in DLP and SLA may require further validation via clinical tests. In contrast, FDM is a commercial AM method that melts thermoplastics into the molten state, in which objects are fabricated using a layer-by-layer approach (Fig. 1a). A significant advantage of the FDM-based method for MNs is that polylactic acid (PLA) can be used to fabricate PLA needle arrays. PLA is a Food and Drug Administration-approved biocompatible material for the application of bone implants, sutures, sustained drug delivery, and vaccine antigens^14^. High molecular weight PLA can be degraded into low molecular weight PLA and biodegradable lactic acid via ester linkage hydrolysis in vivo^15^. Accordingly, PLA is considered safe for use as an MN material. Furthermore, disposal methods for PLA objects include composting, chemical recycling, and mechanical recycling^16^.

The printing resolution of the conventional FDM process cannot produce MNs with a tip diameter of <100 µm, which is the conventional dimension for MNs^17^. To address the limitations of conventional FDM technology, the hydrolysis of PLA was adopted as a chemical etching process to minimize the size of the PLA needles^18–20^. Under alkaline conditions, extra hydroxide ions can accelerate hydrolysis on the surface of PLA (Fig. 1c), which is facilitated by the nucleophilic attack of hydroxide ions on the carbonyl^21,22^. These hydroxide ions can be considered catalysts during the hydrolysis of PLA^23^.

For the fabrication process by FDM and chemical etching to produce PLA MNs, the bonding between the PLA layers is critical. The anisotropic properties of the extruded PLA layers cause weak bonding between the layers. Weak bonding can lead to the detachment of PLA needles from the substrates during the etching process. Localized heating on the predeposition layer by a diode laser can be used to enhance the interfacial bonding strength. However, negative effects, such as hole formation and breakage induced by the laser, can produce defects^24^. Yin et al. applied the intermolecular diffusion theory to predict the polymer interfacial bonding strength during FDM processing^25^. The temperature of the printing stage significantly improves the bonding strength at the interfaces. Nevertheless, the influence of parameters during the chemical etching process is relatively unknown.

In this study, to facilitate welding on the interface of successive PLA layers, we investigated the optimal thermal parameters of the FDM process, such as the nozzle (*T*_0_) and printing stage (*T*_∞_) temperatures. The conditions for the chemical etching process, such as the etchant concentration, environmental temperature, and stirring ratio of the etchant, were investigated to improve the chemical etching efficiency. As illustrated in Fig. 1, to weld the interfaces of successive PLA layers, PLA needles were initially printed using the FDM printer under optimized conditions. The PLA needles were subsequently chemically etched to reduce the dimensions of the PLA needles to reach the micron scale. The fabricated PLA MNs could be coated with APIs for medical applications. This study optimizes the FDM-based fabrication process for the batch fabrication of PLA MNs with preprogrammable structures.

## Results

### MNs fabrication via FDM and chemical etching

The PLA needle array with predesigned structures was printed using an FDM printer (Fig. S1). Two types of PLA needles were used in this study, with needle lengths and layer diameters of 1 and 0.7 mm, respectively. The main difference between the two types of needles was the inclination angle. The first needle type was a straight needle. The second was an inclined needle with an inclination angle of 45°. All samples were printed with an infill density of 100% nominally to ensure that they were optimally solid. Parameters, such as the layer thickness, line width, and printing speed, depend on the function of the FDM printer. Therefore, these parameters were set to the preferred setting of the FDM printer to guarantee the quality of the printed PLA objects. Different thermal parameters, such as *T*_0_ and *T*_∞_, were adjusted to investigate their influence. PLA needles were printed at low thermal parameter values (*T*_∞_ = 60 °C and *T*_0_ = 190 °C), as well as high thermal parameter values (*T*_∞_ = 120 °C and *T*_0_ = 210 °C).

**Fig. 2:**
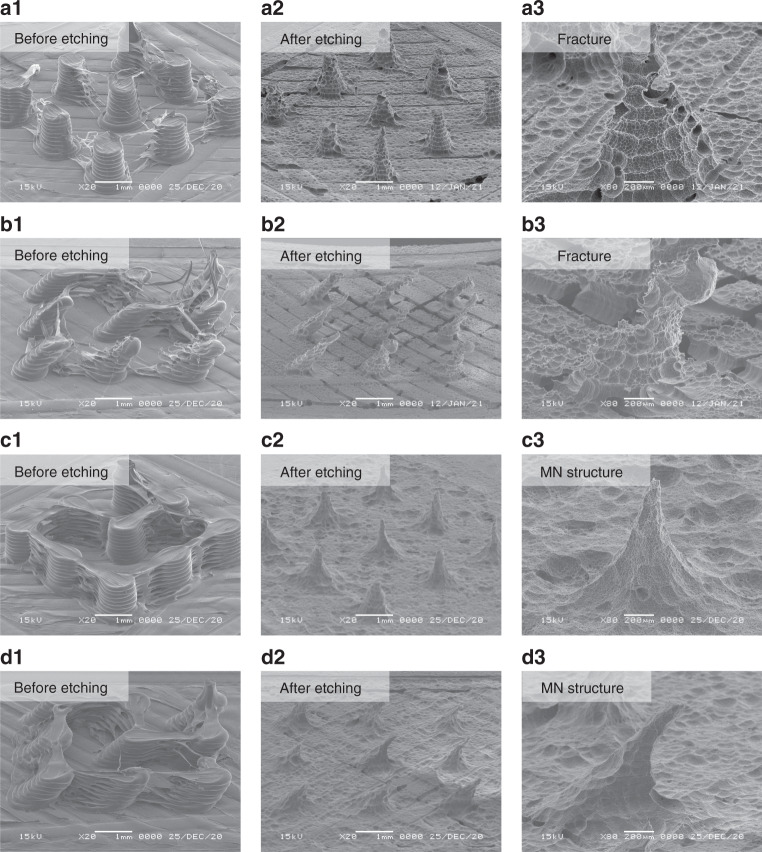
Chemical etching on PLA needles printed at different thermal parameters. **a1**–**a3** Chemical etching on straight PLA needles printed at low thermal parameters (*T*_∞_ = 60 °C and *T*_0_ = 190 °C). **b1–b3** Chemical etching on inclined PLA needles printed at low thermal parameters (*T*_∞_ = 60 °C and *T*_0_ = 190 °C). **c1**–**c3** Chemical etching on straight PLA needles printed at high thermal parameters (*T*_∞_ = 120 °C and *T*_0_ = 210 °C). **d1**–**d3** Chemical etching on inclined PLA needles printed at high thermal parameters (*T*_∞_ = 120 °C and *T*_0_ = 210 °C)

The main difference in the morphology of the PLA needles printed at different thermal parameters was the strings between the PLA needles, as shown in Fig. 2. The stringing phenomenon is a conventional challenge in the FDM process^26^. The strings result from plastic residues on the tip of the printing nozzle. In particular, strings are formed when the nozzle travels from one point to another. This is because the higher thermal parameters trigger a longer duration of the molten state of the deposited PLA. The stringing phenomenon was more significant with a higher flowability of the deposited PLA. The PLA strings were removed by a subsequent etching process, as shown in Fig. 2. Chemical etching was performed in a 1 M sodium hydroxide (NaOH) solution at 55 °C. The stirring speed of the etchant was set to 0 rpm throughout the entire process. The interfacial bonding of the printed PLA needles was significant during the etching process. Fractures on the PLA needles printed at low thermal parameters occurred after etching for 10 h (Fig. 2a3, b3). In contrast, the substrates of the PLA needle array tended to collapse upon further etching owing to the high void density between the PLA layers. Consequently, the PLA needles detached from the substrate during subsequent etching. Therefore, MN structures cannot be obtained from PLA needles printed with low thermal parameters. However, PLA needles with high thermal parameters did not exhibit fractures during the etching process. Owing to the inclination angle, the diameter of the inclined needle was less than that of the straight needle. Straight MN structures were formed after etching for 30 h (Fig. 2c3). Inclined MN structures were formed after 18 h of etching (Fig. 2d3). Both straight and inclined MNs had a needle length of approximately 1000 µm. The straight and inclined MNs had average tip diameters of 97 ± 21 and 86 ± 23 µm, respectively. The inclination angles of the MNs after chemical etching agreed well with the predesigned angles. Therefore, only PLA needles printed at high thermal parameters can be used for subsequent chemical etching in the fabrication of MN structures.

### Evaluation of PLA MNs

**Fig. 3:**
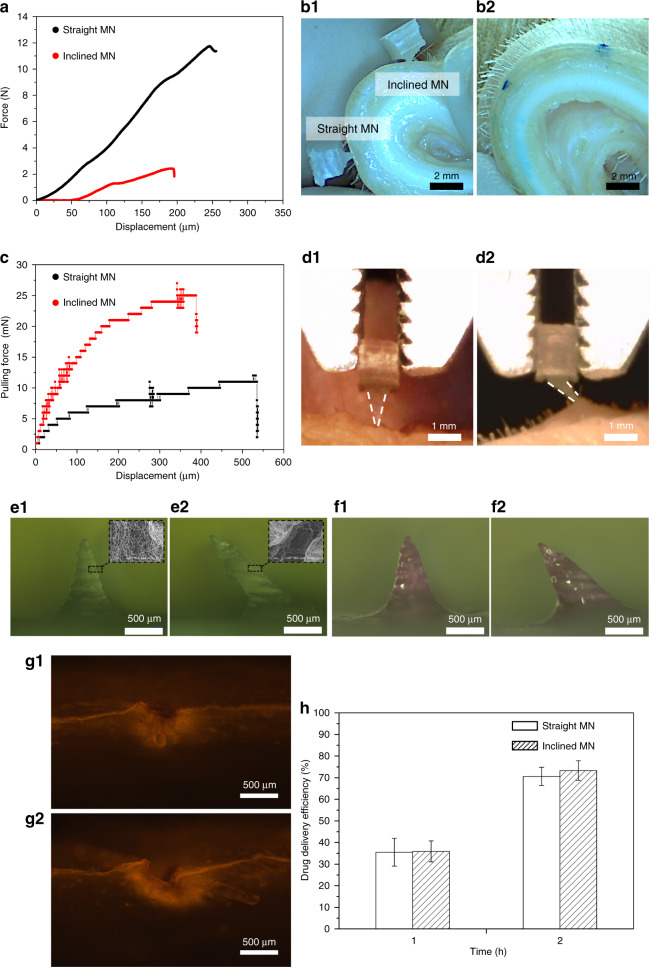
Evaluation results of the fabricated PLA MNs. **a** Strength tests of straight and inclined MNs. **b1** Porcine skin inserted by straight and inclined MNs. **b2** Skin sample stained by methylene blue. **c** Pulling force of straight and inclined MNs from the skin samples. The resolution of the force sensor is 1 mN. **d1** Skin adhesion tests of straight MNs. **d2** Skin adhesion tests of inclined MNs. **e1** Straight MN before coating. **e2** Inclined MN before coating. **f1** Straight MN after coating. **f2** Inclined MN after coating. **g1** Fluorescence image of skin inserted by coated straight MNs. **g2** Fluorescence image of skin inserted by coated inclined MNs. **h** Drug delivery efficiency of the model drug from MNs to the skin, *n* = 3

Strength tests were performed on both straight and inclined MNs to evaluate the PLA MNs realized in this study. The average failure force of straight MNs was 10.5 ± 1.3 N. In contrast, the average failure force of the inclined MNs was 4.8 ± 2.2 N. Representative force–displacement curves of straight and inclined MNs are shown in Fig. 3a. The set-up of the strength tests is shown in Fig. S2. The results indicate that both straight and inclined PLA MNs have sufficient strength (>58 mN) for insertion into skin^27^. Furthermore, both straight and inclined MNs (a single MN on each patch) were inserted into porcine skin, as shown in Fig. 3b1. The results obtained from skin staining with methylene blue (Fig. 3b2) confirmed that both straight and inclined MNs pierced the stratum corneum of the skin samples. After the MNs were inserted into the skin, force–displacement equipment was used to pull the MNs from the skin. The average pulling-out forces of individual inclined and straight MNs were 29.7 ± 3.1 and 15.7 ± 3.2 mN, respectively. The representative pulling force–displacement curves of straight and inclined MNs are plotted in Fig. 3c. Inclined MNs triggered more significant deformations of the skin sample than straight MNs (Fig. 3d1) when pulled from the skin (Fig. 3d2). This result indicates that the adhesion of the inclined MNs was stronger than that of the straight MNs, which agrees with a previous study^3^. Carboxymethyl cellulose (CMC) loaded with 0.02 mg rhodamine B was coated on the surface of each PLA MN (Fig. 3e1, e2) to prepare the coated MNs (Fig. 3f1, f2). The porous morphology of PLA MNs after chemical etching could possibly facilitate drug coating.

In vitro transdermal drug delivery was conducted by inserting straight and inclined MNs coated with rhodamine B into porcine skin for 30 min. After the MNs were removed, the skin samples were sliced. Subsequently, the skin sections were observed under a fluorescence microscope. The heights of both the straight and inclined MNs were approximately 1 mm. Fluorescence images (Fig. 3g1, g2) show significant breaches on the stratum corneum. Furthermore, the fluorescence components reached a depth of approximately 500 µm from the surface of the skin. Therefore, both the straight and inclined MNs penetrated the stratum corneum and epidermis. In addition, owing to its elastic properties, skin can be deformed when pressure is applied. Therefore, the entire shaft of the MN cannot be completely inserted into the skin.

To determine the drug delivery efficiency of the coated PLA MNs, both coated straight and inclined MNs were inserted into porcine skin for certain durations. The mass of rhodamine B (model drug) remaining on the MNs was determined using a microplate reader. As shown in Fig. 3h, after insertion into skin for 1 h, both the straight and inclined MNs delivered 35% of the model drug into the skin. Furthermore, the drug delivery efficiency of MNs reached 75% after insertion for 2 h.

## Discussion

### Physical characteristics of PLA

**Fig. 4:**
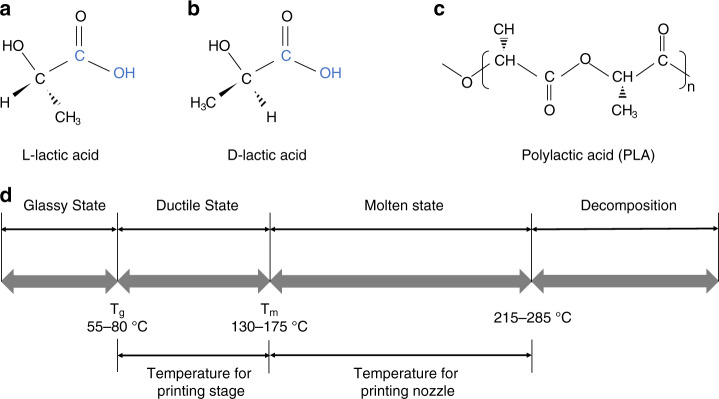
Physicochemical properties of PLA. **a** Chemical structure of l-lactic acid. **b** Chemical structure of d-lactic acid. **c** Configuration of PLA. **d** Metastable states of PLA

The repeat unit of the general PLA contains a stereocenter that is either l- or d-lactic acid, as illustrated in Fig. 4a–c. Commercially available PLA for practical FDM processing comprises a mixture of l- and d-lactic acids but with a major proportion of l-lactic acid. The stereochemistry of PLA is complicated owing to its chiral nature. PLA can be semicrystalline with components of >93% l-lactic acid, whereas components of l-lactic acid ranging from 50 to 93% can make PLA strictly amorphous^28^. The reported molecular weight of PLA for FDM is 50–140 kDa, which is required to stabilize the viscosity of molten PLA during the extrusion process^29,30^.

The relationship between the metastable states of high molecular weight PLA and temperature is shown in Fig. 4d. The glass transition temperature (*T*_g_) indicates the transition between the glassy and ductile states. Temperatures above *T*_g_ can induce drastic changes in the polymer chain mobility. For PLA, *T*_g_ can be represented by the Flory–Fox equation^31^:

1 $$T_{\rm{g}} \approx T_{\rm{g}}^\infty - K/M_{\rm{n}}$$

where $T_{\rm{g}}^\infty$ represents the *T*_g_ of the number-average molecular weight (*M*_n_) and *K* is an empirical constant that indicates the excess free volume for the end groups of the polymer chains. Jamshidi et al. demonstrated that the *T*_g_ of d,l-lactic acid (PDLLA) is higher than that of poly l-lactic acid. The reason for this difference is partly due to the crystallinity of the two polymers^31^. Therefore, the *T*_g_ of PLA adopted for FDM is not a universal constant. Du et al. reported that the *T*_g_ of PLA ranges from 55 to 80 °C^32^.

The melting temperature (*T*_m_) of PLA depends on the presence of PDLLA in the structure and is given by^16^:

2 $$T_{\rm{m}} \approx 175 - 300W_{\rm{m}}$$

where *W*_m_ represents the fraction of PDLLA in the structure. The melting point of PLA composed of 100% l-lactic acid is 175 °C. The *T*_m_ of a typical PLA ranges from 130 to 175 °C^16^.

When PLA is heated above *T*_m_, it rapidly loses its thermal stability. Moreover, significant molecular weight degradation occurs when heating lasts for a long period at a temperature of 10 °C above the melting point. Migliaresi et al. reported that the thermal degradation of PLA is due to chain splitting^33^. Amorphous PLA decomposes between 215 and 285 °C^16^. During FDM processing, the stage temperature of the FDM printer should be set between *T*_g_ and *T*_m_ to ensure that the bottom layer is firmly adhered to the stage. The printing nozzle should be set above *T*_m_ but below the decomposition temperature for the smooth extrusion of PLA. For the PLA filament employed in this study, *T*_g_ and *T*_m_ were 60 and 170 °C, respectively. Furthermore, the decomposition temperature of the PLA filament is 220 °C (Table 1).

**Table 1:** Thermal properties of the PLA filament and PLA processing parameters.

	Value
Thermal properties of PLA	
Thermal conductivity, *k* (W/m·K)	0.13
Specific heat, *C* (J/kg·K)	1800
Density, *ρ* (kg/m^3^)	1240
Glass transition temperature, *T*_g_ (°C)	60
Melting temperature, *T*_m_ (°C)	170
Decomposition temperature, *T*_d_ (°C)	220
Parameters during FDM printing	
Infill density (%)	100
Printing speed, *v* (mm/s)	60
Layer thickness, *L*_t_ (µm)	100
Line width, *L*_w_ (µm)	700
Nozzle temperature, *T*_0_ (°C)	190–210
Printing stage temperature, *T*_∞_ (°C)	60–120

### AM of PLA needles

**Fig. 5:**
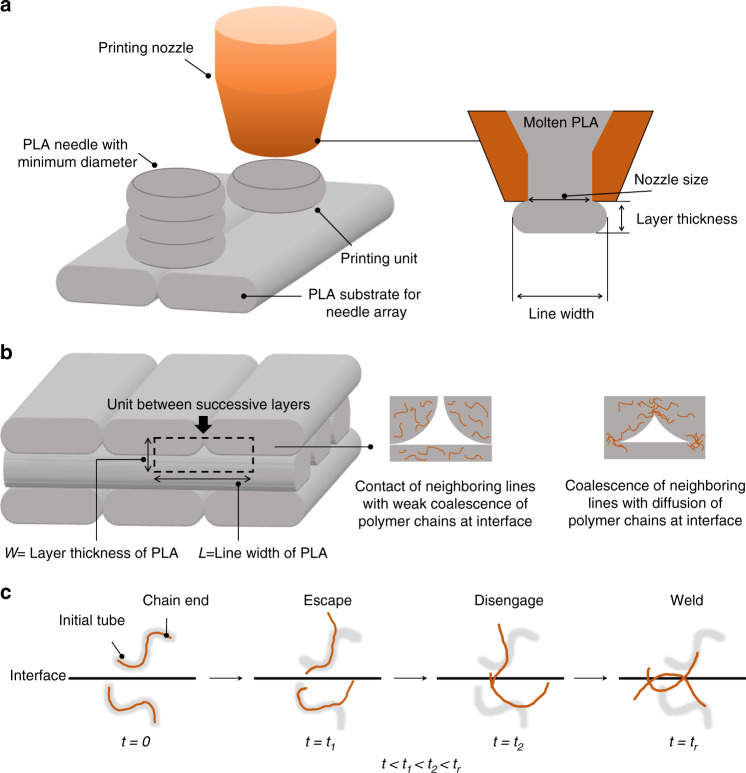
Illustration of PLA objects printed by the FDM printer. **a** The dimension of the printing unit is limited by the nozzle size. **b** The cross-sectional structure of the FDM-printed PLA objects comprises bonded PLA lines and voids between successive layers. **c** Reptation model depicting the behavior of PLA molecular chains during the interfacial diffusion process, where *t*_r_ represents the tube relaxation or disengagement time

The molten PLA extruded from the nozzle can fabricate objects layer by layer in the FDM. Before the hardening and solidification of the previous layer, the polymer chains of PLA diffuse across the interfaces between successive layers to form bonds. When the FDM is adopted to print objects with micron-scale structures, the printable dimensions depend on the printing unit of the FDM printer. The PLA needles were accumulated by the printing units extruded from the nozzle, as illustrated in Fig. 5a. The profile of the printing unit has an oblong shape, which is influenced by two parameters: line width and layer thickness. Herein, the minimum diameter of the printing unit, which is termed “line width”, is limited by the size of the nozzle. In this study, a nozzle with a diameter of 500 µm was used for printing. In addition, the PLA disks deposited after solidification are expected to have diameters >500 µm, considering the thermal deformation of the heating nozzle.

Dimensional error is a conventional challenge during the FDM process, which triggers nonuniformity in the diameters of the deposited PLA disks. Zhang et al. reported that an optimized combination of parameters, such as the line width, layer thickness, and printing speed, can reduce the dimensional error during the FDM printing process^34^. However, the optimized parameter combination varies between FDM printers. In contrast, the positions of the printing nozzle and printing stage can be controlled to the required layer thickness by fixing the gap distance between the nozzle and stage. Similar to the initial layer, the thickness of each layer was controlled. Accordingly, the length of the printed PLA needles can also be controlled.

During the layer-by-layer deposition process, the layers were composed of partially bonded PLA lines and voids between two successive layers, as illustrated in Fig. 5b. Until the PLA was cooled below the glass transition temperature, the mobility of the polymer chains inside the extruded PLA was activated^35^. The mobility of the polymer chains continuously facilitates coalescence between neighboring PLA lines. Furthermore, the PLA in the molten state exhibited better flowability than that in the ductile state. The conventional reptation model was used to describe the motion of random coil chains at the interfaces of the molten polymer within the relaxation time, as illustrated in Fig. 5c. The PLA molecular chain near the interface is initially confined in a tube, which is a topological constraint that restricts the motion of the chain. Brownian motion enables the chain ends to escape from the initial tubes as random coil chains obeying Gaussian statistics^36^. The PLA random coil chains diffuse across the interface of neighboring PLA lines and entangle PLA chains from the other side of the interface to weld the interface. The number (*n*) of random coil chains diffusing across the unit area of the interface can be expressed by Eqs. 3 and 4:

3 $$n(t) \approx t^{1/4}M^{ - 5/4};(t\, < \,t_{\rm{r}})$$

4 $$n_\infty \approx M^{ - 1/2};(t \ge t_{\rm{r}})$$

where *t* and *M* represent the contact time of successive molten PLA lines and molecular weight of the polymer chains, respectively. In addition, *t*_r_ represents the relaxation time of the polymer chain, and in the dynamic polymer solution, *t*_r_ ≈ *M*^3 ^^36^.

This indicates that the interfaces of successive PLA layers can be welded to a virgin state by contacting the molten PLA depositions until the relaxation time. However, during the FDM deposition process, the two layers remain in the molten state for a limited time owing to natural cooling. A conventionally accepted analytical solution by Bellehumeur et al. uses a lumped capacity analysis to predict the cooling time of the extruded layer during the FDM process^37,38^:

5 $$t = \frac{{ - \ln \left( {\frac{{T - T_\infty }}{{T_0 - T_\infty }}} \right)}}{{mv}}$$

where *T* and *T*_∞_ represent the transient temperature of the extruded PLA layer during cooling and envelope temperature around the extruded PLA layer, respectively. *T*_∞_ was assumed to be consistent with the printing stage temperature because the printed PLA needle patch in this study was relatively thin.*T*_0_ represents the extrusion temperature of the PLA, which is the same as the nozzle temperature, and *v* is the constant moving speed of the nozzle during the printing of one layer. Furthermore, $m = \frac{{\sqrt {1 + 4\alpha \beta } - 1}}{{2\alpha }}$, $\alpha = \frac{k}{{\rho Cv}}$, and $\beta = \frac{{hP}}{{\rho CAv}}$, where *A* and *P* represent the cross-sectional area and perimeter of the extruded PLA, respectively. In addition, *h* is the free convection from the extruded PLA to the envelope air, which is estimated to be 50–100 W/m^2^·K, while *ρ*, *k*, and *C* represent the density, thermal conductivity, and specific heat of the PLA, respectively.

The reptation model assumes that the contacting polymers are in a molten state. Equation 5 can be adopted to predict the cooling time between the extrusion and melting temperature. For the *T* = *T*_*m*_ case, increasing both *T*_∞_ and *T*_0_ can extend the diffusion time of PLA chains at the interfaces before cooling below *T*_m_. The extended diffusion time at the interfaces of the PLA layers can facilitate bonding between successive layers, as expressed by Eq. 3. Notably, the profile of the extruded PLA lines and printing speed depend on the functions of the FDM printer. These two parameters significantly influence the quality of the printed objects. *A*, *P*, and *v* were set as the preferred settings for the FDM printer. Therefore, they are constant in Eq. 5.

Inadequate contact between neighboring PLA lines triggers the formation of voids between layers. A rectangular unit between two successive layers is determined by the line width and layer thickness of PLA. The void density in the area of the rectangular unit was measured via microscopy and analyzed using the ImageJ software.

Equation 5 indicates that the cooling time until the solidification of PLA is linear with a value of $- \ln \left( {\frac{{T_{\rm{m}} - T_\infty }}{{T_0 - T_\infty }}} \right)$. If the other parameters are constant, then different combinations of *T*_0_ and *T*_∞_ can trigger different bonding performances between the contacted PLA layers. With respect to the FDM printer used in this study, the maximum *T*_∞_ is 120 °C. Therefore, *T*_∞_ was set to the range 60–120 °C during the printing process. In addition, the nozzle temperature was kept below 210 °C to avoid decomposition of the PLA. Based on the thermal properties of the PLA filament in this study, the printing nozzle temperature of the FDM was set to 190–210 °C during the printing process.

### Coalescence of PLA layers

**Fig. 6:**
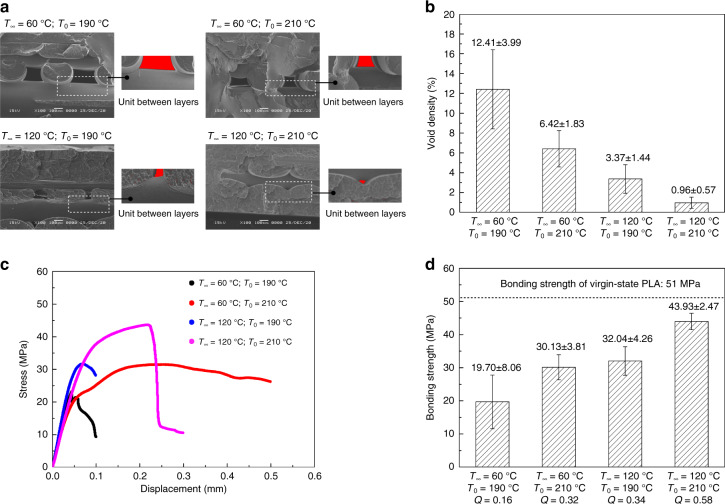
Coalescence of PLA layers at different nozzle and printing stage temperatures. **a** SEM images of cross-sectional views of PLA substrate printed under different conditions. **b** Average void density of PLA substrates printed under different conditions, *n* = 4. **c** Representative stress–displacement curves of uniaxial tensile tests for PLA needles printed under different conditions. **d** Average bonding strength of PLA needles printed under different conditions, $Q = - \ln \left( {\frac{{T_{\rm{m}} - T_\infty }}{{T_0 - T_\infty }}} \right)$ and *n* = 6

The fractured surfaces of representative samples printed with different combinations of *T*_0_ and *T*_∞_ are presented in Fig. 6a. Although the nominal infill density was set to 100%, the cross-sections of the PLA substrates still exhibited voids between successive layers. The results in Fig. 6b indicate that the void density can be reduced as both *T*_0_ and *T*_∞_ increase. If *T*_∞_ is increased, then the void density can be effectively reduced with a constant *T*_0_ value. Because the printing stage was heated above the glass transition temperature of PLA, the PLA in the ductile state filled the internal voids under the influence of gravity. This was because the PLA needles accumulated by the deposition of the printing units. The challenge of voids inside needle structures can be ignored. The bonding strength of the successive PLA layers of the PLA needles was investigated by pulling the PLA needles from the substrate using force–displacement equipment. Equations 3 and 5 indicate that the bonding performance of successive PLA layers is positively correlated with the value of $- \ln \left( {\frac{{T_m - T_\infty }}{{T_0 - T_\infty }}} \right)$, which is herein defined as *Q*. The numerical results of the bonding strengths measured by tensile tests, as presented in Fig. 6c, d, agree well with the corresponding *Q*. Furthermore, the measured tensile strength of the PLA filament in the virgin state was approximately 51 MPa using the force–displacement machine. The experimental data indicated that the interfaces of successive PLA layers could be welded. The bonding strength of the PLA needles increased from 19.70 MPa (*Q* = 0.16) to 43.93 MPa (*Q* = 0.58) as *T*_0_ and *T*_∞_ increased from their minimum to maximum values. In this study, the welding of the layer interface could not reach the virgin state of PLA with the maximum *T*_0_ and *T*_∞_. An optimized bonding strength of PLA needles is necessary to avoid detachment during the chemical etching process.

Yin et al. reported that optimizing the nozzle temperature or printing stage temperature can improve the interfacial bonding strength of FDM-printed objects^25^. In this study, a polymer reptation model and prediction model for the cooling time of a polymer extruded by FDM were employed. The combination of these two models offers universal optimization for the entire FDM process. Interfacial bonding was mutually influenced by *T*_0_ and *T*_∞_. The void density and interfacial bonding between successive PLA layers are critical in subsequent chemical processes. PLA needles with weak interfacial bonding and high void density will trigger detachment of the PLA needles and collapse of the substrate during etching, resulting in production failures and waste of raw materials.

### Chemical etching process

**Fig. 7:**
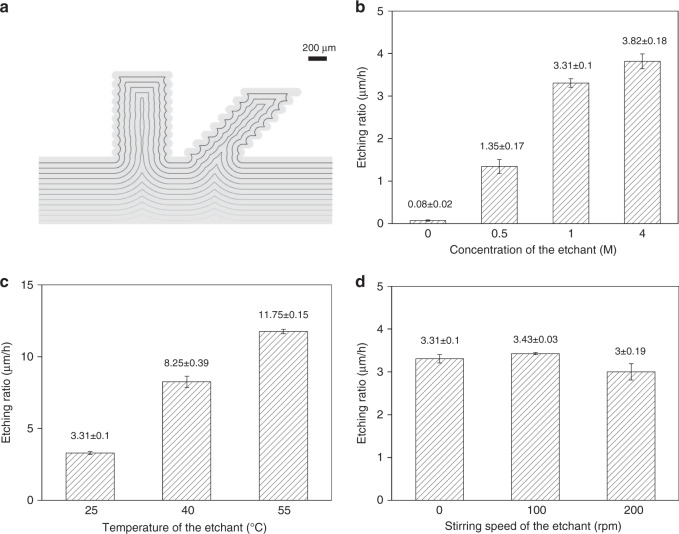
Characteristics of the chemical etching process. **a** Simulation of the transformations of PLA needles during the chemical etching process. The etched amount of each step in the simulation is approximately 70 µm. **b** Etching ratios of PLA needles in NaOH solution at different concentrations. The temperature is 25 °C, and the etchant stirring speed is 0 rpm for each condition. **c** Etching ratios of PLA needles in 1 M NaOH solution at different temperatures. The stirring speeds of the etchants are all set to 0 rpm. **d** Etching ratios of PLA needles in 1 M NaOH solution at different stirring speeds. The temperatures of the etchants are all set to 25 °C

The concept of combining FDM processing and chemical etching to fabricate PLA MNs has previously been proposed^18,19^. However, the bonding limitations of PLA layers during FDM processing have yet to be addressed. The PLA needles printed via FDM have uneven profiles owing to the layer-by-layer deposition process. The simulation of the transformation of PLA needle structures during the etching process demonstrated that barbed profiles could be smoothed with isotropic etching on the bulks of the needle patches (Fig. 7a). Furthermore, the chemical etching of PLA needles printed with different layer thicknesses was simulated (Fig. S3) and experimentally investigated (Fig. S4). The results indicated that the barbed PLA MNs reported by Luzuriaga et al. were potentially due to inadequate etching^19^.

A PLA needle array printed with *Q* = 0.58 (*T*_∞_ = 120 °C and *T*_0_ = 210 °C) was chemically etched (Fig. S5a). The curves of the etched amount within 20 h were fitted linearly (Fig. S5b–d) to obtain PLA etching ratios. The etching ratios of PLA under different conditions were measured and are presented in Fig. 7b–d. As shown in Fig. 7b, the etching ratio increased with the concentration of the etchant. Furthermore, once the concentration of hydroxide ions reached 1 M (pH = 14), increasing the concentration of the etchant was ineffective in accelerating the etching ratio. This result can be attributed to the saturation of hydroxide ions inside the solvent. In addition, it was evident that the high temperature of the etchant significantly accelerated the etching ratio, as shown in Fig. 7c. This result can be explained by the theory of thermodynamics, which indicates that a higher temperature promotes the diffusion of molecules. Water molecules, together with hydroxide ions, need to penetrate and diffuse into the PLA polymer matrix to break the ester bones. Increasing the temperature accelerated the hydrolysis of PLA. However, the stirring speeds of the etchants did not significantly influence the etching ratio, as shown in Fig. 7d. This result agrees well with that of a previous study by Román-Ramírez et al. It has been reported that the catalyst-mediated hydrolysis of PLA does not exhibit mass-transfer limitations owing to stirring^23^.

## Conclusion

We investigated an optimized methodology for the fabrication of PLA MNs with tailored structures. A parametric study of the influence of PLA layers on the interfacial bonding strength was conducted by investigating the nozzle and printing stage temperatures of the FDM process. Within the temperature range that the FDM printer and PLA filament can withstand, increasing the thermal parameters during the FDM process was demonstrated to improve the interfacial bonding strength and decrease the void density of the PLA layers. The optimized PLA needle array can effectively prevent detachment of the PLA needles and collapse of the substrates during etching. This study suggests that increasing the concentration and temperature of the etchant can improve the efficiency of chemical etching. Based on these findings, a customized methodology was developed for the fabrication of biodegradable MNs. Our study is expected to provide the MN field with a method for fabricating MN structures for various biomedical applications without expertise in microfabrication.

## Materials and methods

### FDM printing of PLA needles

The PLA needles in this study were printed using an FDM printer (Taz 6, Lulzbot, USA) in a layer-by-layer manner prior to chemical etching. The geometrical structures of the printing objects were designed using the Fusion 360 software and converted into G-code using the Cura software, which can be recognized by the FDM printer for printing. The thermal properties of the commercial PLA filament (16609, Mutoh, Japan) and printing parameters employed in this study are listed in Table 1.

### Chemical etching by hydrolysis

The degradation of PLA can be considered a post-fabrication method to reduce the size of FDM-printed PLA needles. There are four main irreversible degradation mechanisms of PLA: photodegradation, microbial degradation, enzymatic degradation, and hydrolysis^39^. In the case of hydrolysis, water molecules break the ester bonds of PLA, which constitute the polymer bones. Degradation can homogeneously act on the PLA surface, which is an essential criterion for postprocessing FDM-printed PLA needles. Therefore, the hydrolysis of PLA was used for chemical etching in this study.

The dimensions of the PLA needles during the etching process were simulated by the MATLAB software using the function “distance transform”. To investigate the influence of different conditions during chemical etching, etchants with concentrations of sodium hydroxide (221465, Sigma, USA) ranging from 0 to 4 M were prepared. A beaker containing 300 mL of NaOH solution was placed on a magnetic stirrer (HS 7, IKA, Japan). The temperature of the etchant was controlled by a heater (temperature in the range 25–55 °C). Furthermore, the stirring speed of the stirring bar inside the beaker was varied from 0 to 200 rpm. The dimensions of the PLA needles before and after chemical etching were measured using an optical digital microscope (VHX-2000, Keyence, Japan). The changes in the diameters in the middle parts of the PLA needles were measured every 5 h after chemical etching. The etched quantities of PLA needles within 20 h were plotted using the Origin software. The curves were linearly fitted to yield the etching ratios under different etching conditions.

### Mechanical tests

The interfacial bonding strength between successive PLA layers is defined as the tensile strength at the interface of the PLA layers. PLA needles printed with different thermal properties were evaluated by uniaxial tensile tests using commercial force–displacement equipment (MX2-500N, Imada, Japan). The grip of the equipment pulled up the PLA needles at a speed of 2 mm/min. The bonding strength (*σ*) of the PLA needles was calculated by:

6 $${\sigma} = \frac{F}{S}$$

where *F* is the stretching force and *S* is the cross-sectional area of the PLA needles.

The strengths of the PLA MNs after chemical etching were measured by compressing the MNs at a speed of 2 mm/min using the force–displacement equipment.

### Evaluation of skin insertion and adhesion

PLA MN array patches were applied to the stratum corneum of porcine ear skin (K1270, Funacoshi, Japan). The criteria for selecting skin samples were based on their similarities with human skin in terms of physiology and anatomy^40^. The puncturing by the PLA MNs was confirmed via a skin staining technique using a 10 mg/mL methylene blue (M9140, Sigma-Aldrich, USA) solution. Methylene blue is a dye used for biological staining, in which dye molecules bind with proteins in tissues (other than the stratum corneum). The methylene blue solution, which has a low molecular weight and is hydrophilic, cannot be absorbed by the hydrophobic stratum corneum^41^. After removal of the PLA MN arrays from the porcine skin, the methylene blue solution was deposited on the skin surface for 10 min. The solution was subsequently wiped away using ethanol (24194, Sigma-Aldrich, USA), leaving the dye only in regions where the stratum corneum had been punctured.

The adhesion performance of the individual PLA MNs was evaluated after skin insertion using the force–displacement equipment. In this study, skin samples inserted with PLA MN arrays were fixed on the stage of the force–displacement equipment. A grip was used to pull the MN arrays out of the skin. The maximum force required to pull out the MN from the skin sample was used to assess adhesion performance.

### Scanning electron microscopic (SEM) characterization

A scanning electron microscope (JSM-6060, JEOL, Japan) was used to observe the PLA objects presented in this study. Prior to observation, a 12-nm-thick layer of gold was coated on the surface using a thin-film coater (SC-701Mk, Sanyu Electron, Japan). Cross-sectional views of the PLA layers were observed using SEM. The void densities were subsequently calculated using the ImageJ software.

### Drug coating

An aqueous solution of 20 mg/mL rhodamine B (83689, Sigma-Aldrich, USA) and 80 mg/mL CMC (C5678, Sigma-Aldrich, USA) was prepared as the coating ingredient. CMC served as the thickener to ensure that the solution was coated on the surface of the PLA MNs^42^. Rhodamine B was used as the model drug in this study. After the MNs were achieved, 1 µL of solution was dispensed on each MN using a micropipette. The coated MNs were heated in an oven at 50 °C for 5 min to solidify the coating ingredients.

### In vitro transdermal drug delivery

Before the test, the skin samples were thawed at 25 °C for 20 min and then completely shaved. Then both coated straight and inclined MN patches were inserted into the porcine ear skin for certain durations. After the MNs were peeled off, the skin samples were sliced. The skin sections were observed using a stereomicroscope (IX71, Olympus, Japan) to obtain fluorescence images.

### Drug loading and drug delivery efficiency

The drug loading and drug delivery efficiency of rhodamine B (model drug) were measured using a microplate reader (H1, BioTek, USA). Rhodamine B in deionized (DI) water at different concentrations (0.5–8 mg/L) created a spectral profile at wavelengths of 400–600 nm using a microplate reader (Fig. S6a). The maximum absorption of light by the chromophore group occurred at a wavelength of 555 nm. When the concentration of rhodamine B ranged from 0.5 to 8 mg/L, the absorbance of light at 555 nm by rhodamine B was linear with the concentration (Fig. S6b). After dissolving one coated PLA MN in 5 mL DI water, the drug loading of rhodamine B on each MN was calculated using the calibration curve. After in vitro transdermal drug delivery tests, each MN was dissolved in 5 mL of DI water. Using the same method, the amount of rhodamine B remaining on each MN after the tests was calculated. Finally, the drug delivery efficiency was calculated.

## Supplementary information

Supplementary information for the paper
