# Supplementary material for: Optimization of the fused deposition modeling-based fabrication process for polylactic acid microneedles
Source: Microsyst Nanoeng. 2021 Aug 2;7:58. doi: 10.1038/s41378-021-00284-9 (PMC8433210; doi:10.1038/s41378-021-00284-9)
Supplement: Supplementary file 1 — Supplementary information for the paper [file 41378_2021_284_MOESM1_ESM.docx]

Supplementary information for the paper:

Optimization of the fused deposition modeling-based fabrication process for polylactic acid microneedles

Libo Wu^1^, Jongho Park^1^, Yuto Kamaki^1^, and Beomjoon Kim^1*^

^1^ Institute of Industrial Science, The University of Tokyo, 4-6-1 Komaba, Meguro-ku, Tokyo 153-8505, Japan

*Corresponding author: Beomjoon Kim (bjoonkim@iis.u-tokyo.ac.jp)

**Supplementary Figures:**


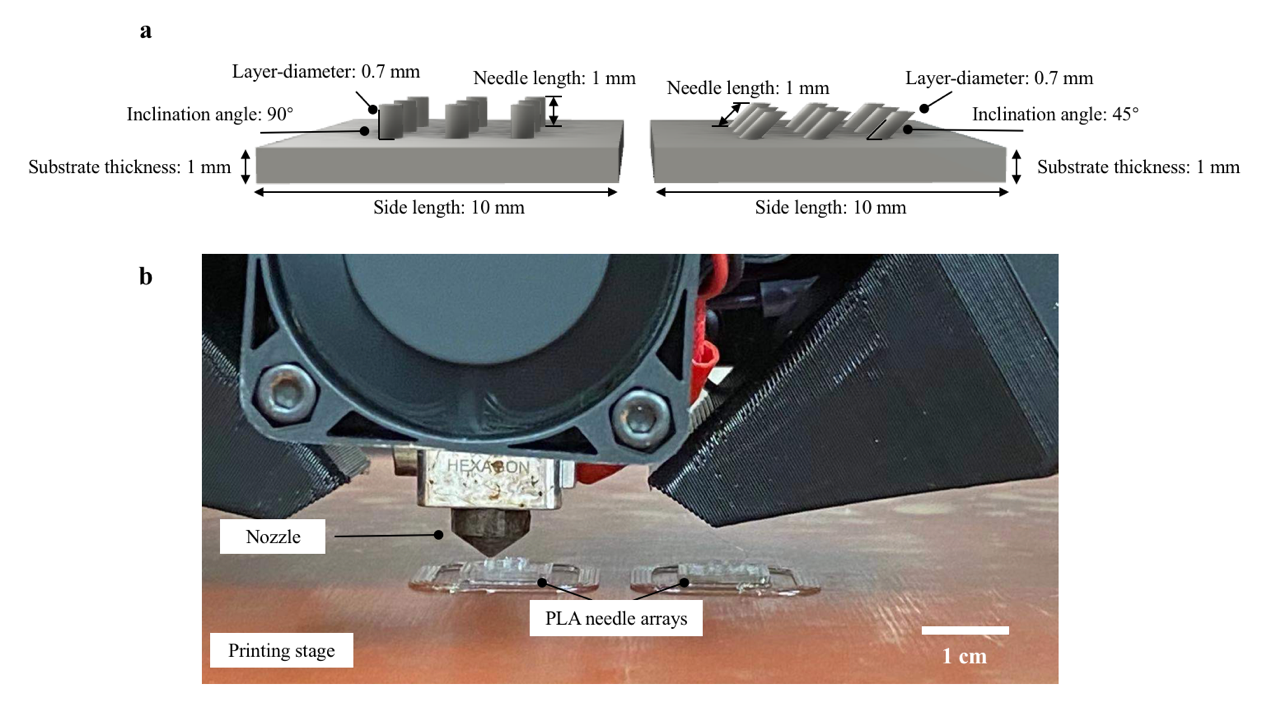


Fig. S1 Fabrication process of PLA needle arrays: (a) design of PLA needle arrays with different inclination angles; (b) FDM printing of PLA needle arrays.


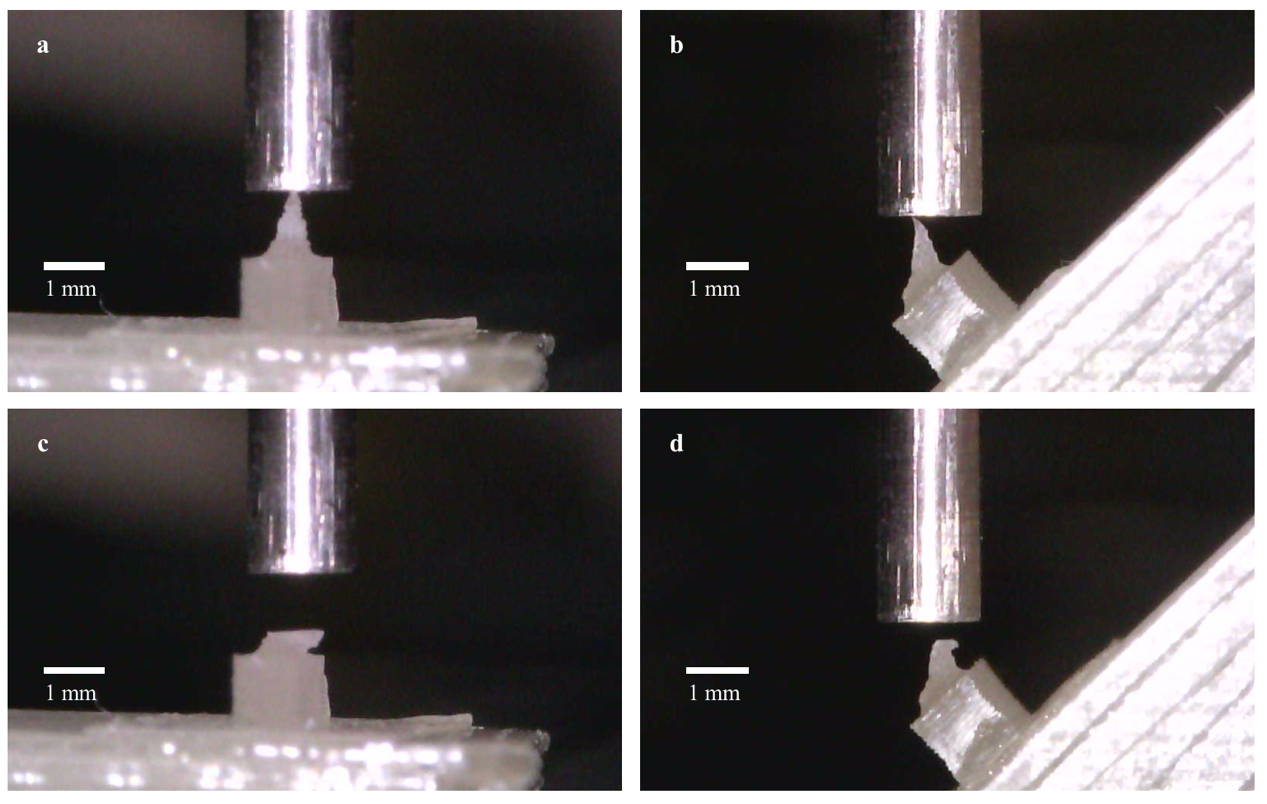


Fig. S2 Setups of strength test. (a) Straight MN before compression. (b) Inclined MN before compression. (c) Straight MN after compression. (d) Inclined MN after compression.


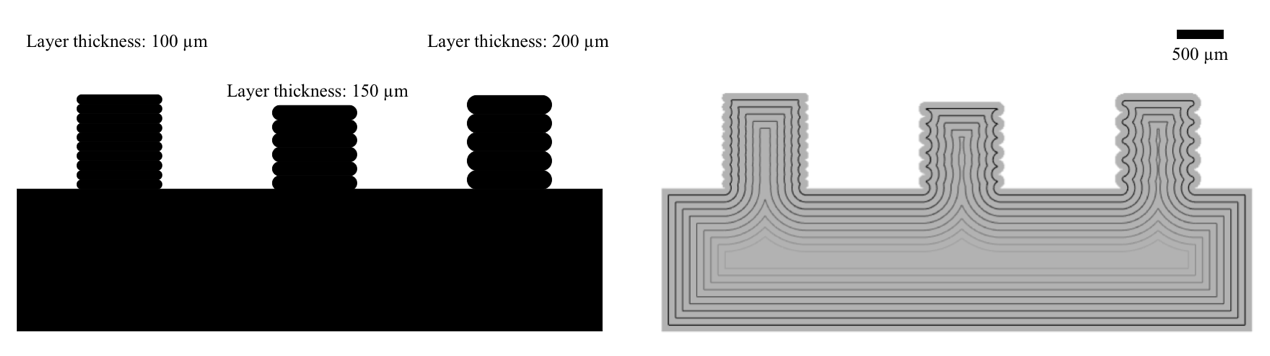


Fig. S3 Transformation of PLA needles during isotropic chemical etching.


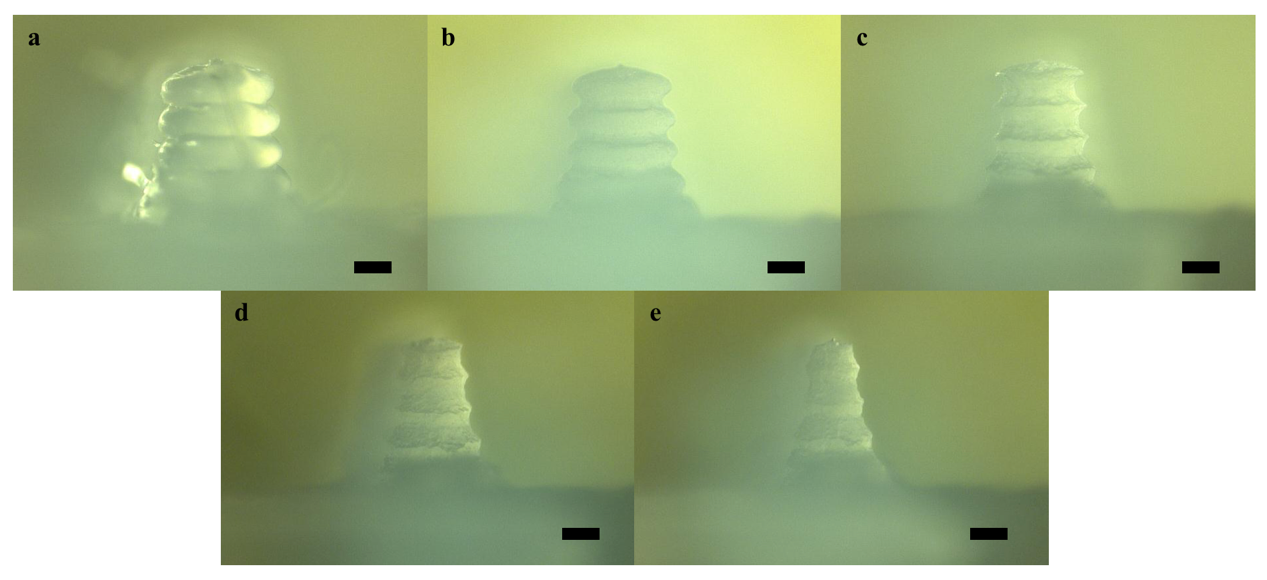


Fig. S4 Transformation of a PLA needle in 1M NaOH solution at 55 °C. The scale bar is 200 µm. (a) PLA needle printed by FDM. (b) PLA needle etched for 5 h. (c) PLA needle etched for 10 h. (d) PLA needle etched for 15 h. (e) PLA needle etched for 20 h.


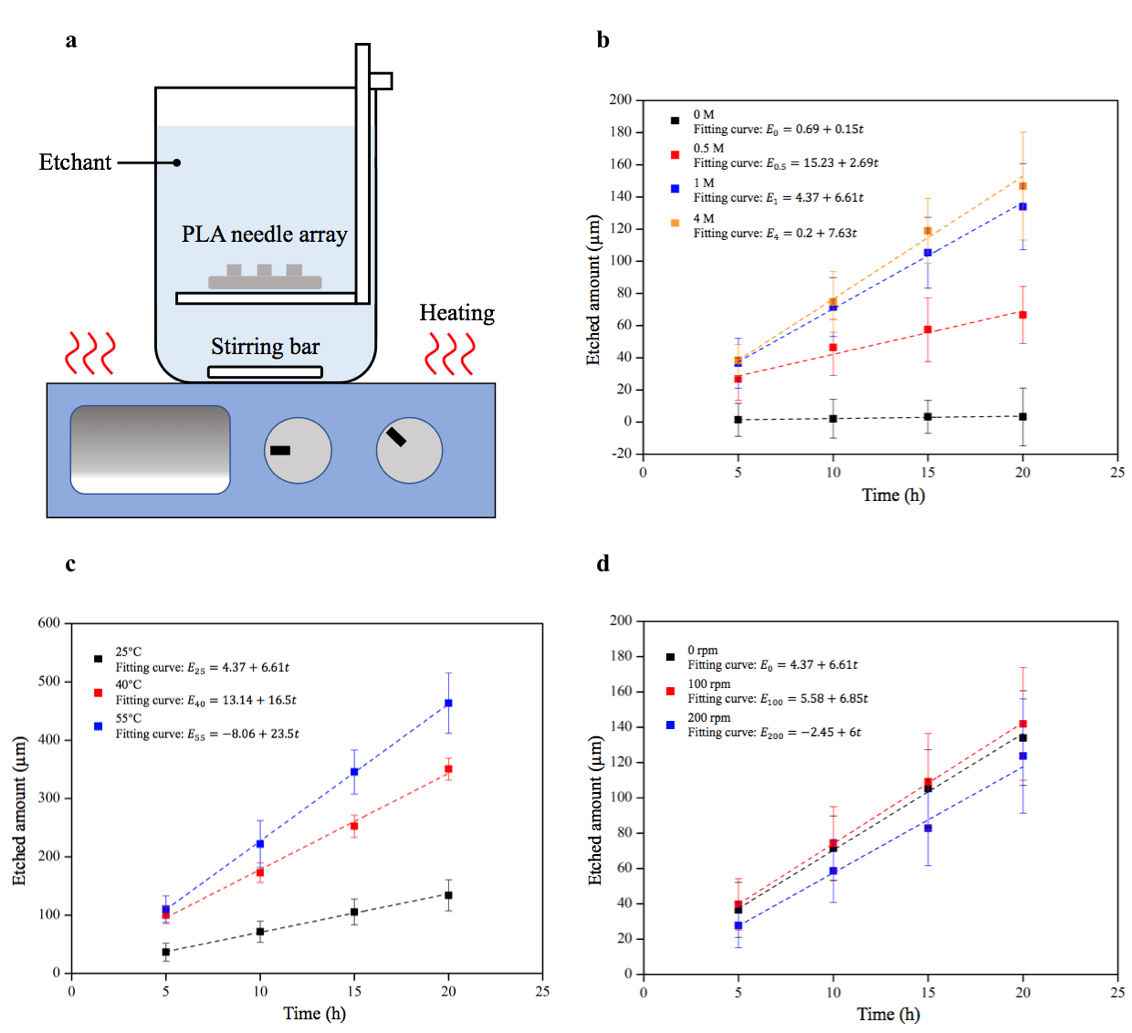


Fig. S5 Chemical etching of PLA needles with different concentrations. (a) Setup of chemical etching. The magnetic stirrer was utilized to heat the beaker consistently and control the temperature of the etchant as well as the stirring speed. (b) The Etched amount of PLA needles in NaOH solution with different concentrations. The temperature is 25 °C and stirring speed of the etchant is 0 rpm for each condition. *n* = 12. (c) The etched amount of PLA needles in 1 M NaOH solution with different temperatures. The stirring speeds of the etchants were all set to be 0 rpm. *n* = 12. (d) The etched amount of PLA needles in 1 M NaOH solution with different stirring speed. The temperatures of the etchants were all set to be 25 °C. *n* = 12.


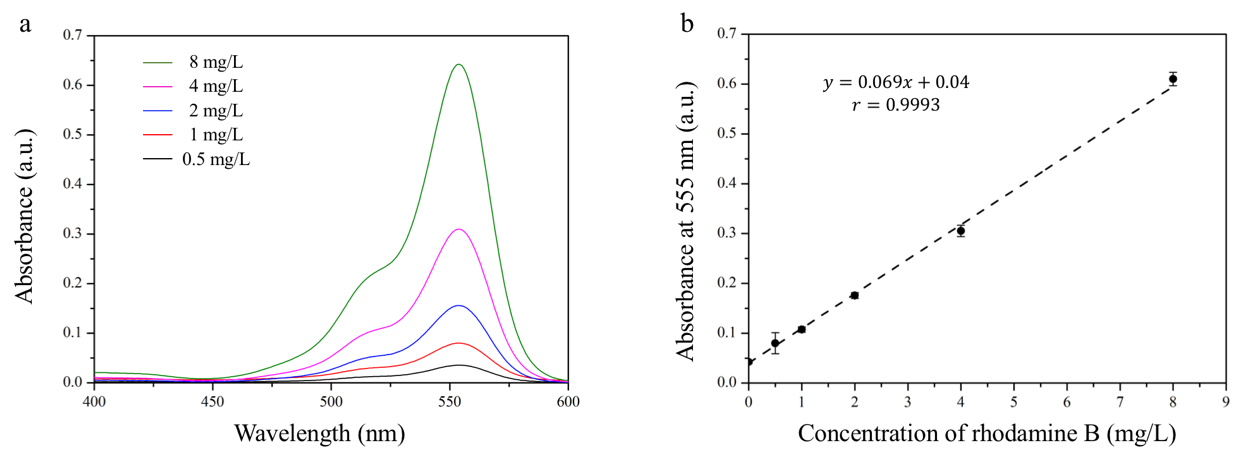


Fig. S6 Calibration of rhodamine B in DI water with different concentrations: (a) the absorbance spectral profile of rhodamine B solution with concentration of 0.5–8 mg/L at 400–600 nm; (b) calibration curve of rhodamine B in DI water with different concentrations.
